# Septic Tenosynovitis of the Digital Flexor Tendon Sheath in 83 Cattle

**DOI:** 10.3390/ani10081303

**Published:** 2020-07-30

**Authors:** Alexandra Hund, Markus Senn, Johann Kofler

**Affiliations:** Department of Farm Animals and Veterinary Public Health, University Clinic for Ruminants, University of Veterinary Medicine Vienna, 1210 Vienna, Austria; alexandra.hund@vetmeduni.ac.at (A.H.); sennmarkus@outlook.com (M.S.)

**Keywords:** digital flexor tendon sheath, tenosynovitis, tendon resection, postoperative survival time, cattle

## Abstract

**Simple Summary:**

Lameness is an ongoing challenge for the cattle industry because it affects the wellbeing and productivity of the animals. Lameness is mostly caused by claw disorders; however, infection of the digital flexor tendon sheath (septic tenosynovitis) is a frequent complication of claw lesions and penetrating wounds located at the lower limb in cattle. Our aim was to describe clinical findings, methods of diagnosis, and outcome in cattle diagnosed with this condition. We aimed to illustrate three different surgical techniques and their success, including the improvement of gait (locomotion) and life expectancy of cattle after surgery. We found that most animals that were subjected to surgical treatment could be discharged cured, even though postsurgical complications had occurred in 17 animals. In all cattle, locomotion improved over the course of hospitalization, which lasted between 13 and 21 days in most cases. After treatment, cattle lived for another 23.7 months on average. This allowed the patients to almost reach the life expectancy of an average Austrian dairy cow. Therefore, we conclude that surgical treatment of cows for septic tenosynovitis of the digital flexor tendon can be performed successfully and is an economically viable option to keep cattle in the herd.

**Abstract:**

Septic tenosynovitis of the digital flexor tendon sheath (DFTS) is the second most prevalent infection of deeper structures of the distal limb in cattle, after septic arthritis of the distal interphalangeal (DIP) joint. Depending on the type of infection and the involvement of adjacent anatomical structures, various surgical techniques may be used for therapy: Incising the DFTS to resect one or both digital flexor tendons (RDFT), additional resection of the DIP joint (RDIP) or additional digital amputation (RAMP). Our goal was to describe clinical findings and outcome in cattle patients (euthanasia vs. treatment) and the success of surgical methods including improvement of locomotion and postoperative survival time (POST). Data of eighty-three cattle with a mean age of 4.3 years were reviewed in this retrospective study. Overall, 57.7% of tenosynovitis cases were in the lateral DFTS of a hind limb. Fifty-five cattle were treated surgically; the remaining 28 cattle were euthanized following diagnosis. The median cumulative POST was 17.3, 83.1, and 11.9 months for RDFT, RDIP, and RAMP, respectively. Fatal postoperative complications occurred in three cattle. We conclude that the applied methods were successful and allowed the animals to almost reach the average life expectancy of an Austrian dairy cow.

## 1. Introduction

Septic tenosynovitis of the digital flexor tendon sheath (DFTS) is the second most prevalent infection of deeper structures of the distal limb in cattle, after septic arthritis of the distal interphalangeal (DIP) joint [1,2,3]. It can arise directly from penetrating wounds, or secondarily from ascending infections from sole ulcers, white-line abscesses or interdigital necrobacillosis [4,5,6]. Hematogenous and iatrogenic infections of the DFTS are rarely seen in cattle [5].

To diagnose and treat tenosynovitis of the DFTS, intimate knowledge of the anatomy of the region is essential. In approximately 1% of cattle, the DFTS communicates with the plantar/palmar recess of the proximal interphalangeal (PIP) joint or the podotrochlear bursa [7]. The two DFTS of a limb do not communicate with each other. However, they are separated only by their synovial capsules that are less than 1 mm in thickness [4,7]. The same thickness of tissue separates the DFTS from the plantar/palmar recess of the fetlock joint at the point distal to the proximal sesamoid bones. These conditions facilitate ascending infections from the claw to the DFTS, as well as from an untreated septic tenosynovitis of the DFTS to the neighboring DFTS or the fetlock joint [4,8,9].

Depending on the route of infection, the enclosed digital flexor tendons (DFT) and adjacent structures such as the distal sesamoid bone, the podotrochlear bursa, parts of the flexor tubercle, the insertion of the deep digital flexor tendon (DDFT) at the distal phalanx, the PIP and DIP joints, the neighboring DFTS, the fetlock joint, and one or more proximal sesamoid bones can all be involved in the infectious process [2,5,10].

Typical clinical signs of unilateral septic tenosynovitis of the DFTS are unilateral swelling from the heel to approximately 8 to 10 cm proximal to the dewclaws and, in some cases, upward tilting of the tip of the claw. The tilting (hyperextension) of the claw is a characteristic indication that the DDFT is no longer intact. This may be caused by traumatic transection or necrosis of the DDFT at any location within the DFTS. In cases of ascending claw infections, the necrosis of the DDFT often occurs at the insertion at the flexor tubercle [3,11].

The main differential diagnoses for clinical symptoms seen in cases of septic tenosynovitis of the DFTS including pain and dilation of the DFTS are traumatic tendinitis of the DFT and desmitis of the suspensory ligament (medial interosseus muscle) with partial or complete rupture of the fibers. These pathological processes can be discerned from septic tenosynovitis using ultrasonography [12].

Septic tenosynovitis of the DFTS is best diagnosed by clinical and orthopedic examination, as well as ultrasonographic examination using a 5–7.5 MHz linear probe [13]. Additionally, the synovial space may be punctured to examine the synovial fluid macroscopically and to collect material for bacterial culture and sensitivity.

Depending on the type of infection of the DFTS (serous, serofibrinous, fibrinous, or purulent) and the degree of involvement of adjacent structures, various surgical techniques may be used for therapy. These include irrigation of the DFTS using large bore needles [3], or an arthroscope [4,14], and opening the DFTS to resect one or both DFT [10,15,16]. Affected soft or bone tissue of adjacent structures must be removed, and occasionally the amputation of the digit becomes necessary to successfully treat and cure the condition [10,11,16].

An important indicator for the medical and economic success of treatment is postoperative survival time (POST) [11,17,18]. Several studies have investigated POST after digital amputation or DIP joint resection but very few have done so for the treatment of septic tenosynovitis of the DFTS [3,19].

The aim of this study was to describe clinical findings, diagnostic approaches, and the outcome in cattle patients affected by septic tenosynovitis of the DFTS. In cattle that were subjected to treatment we aimed to illustrate three surgical methods, their associated postoperative complications as well as their success including improvement of locomotion and POST.

## 2. Materials and Methods

### 2.1. Patients

In this study, we retrospectively analyzed patient information records for cattle with septic tenosynovitis of the DFTS that were treated at the University Clinic for Ruminants of the University of Veterinary Medicine in Vienna from January 2001 to December 2017. Animals were excluded if septic tenosynovitis of the DFTS was a secondary finding and did not influence clinical decision-making. This included cattle with polyarthritis, polysynovitis, animals with other severe comorbidities and animals where the DFTS was only irrigated during digital amputation.

Age, sex, breed, locomotion score (LS) at admission and at discharge, surgical and medical therapy, and the duration of treatment at the clinic were recorded. Time of and cause for culling were evaluated by telephone interviews with the owners of the cattle in December 2018 and time of culling was evaluated again in July 2020.

### 2.2. Clinical Examination and Diagnoses

The following describes the standard procedure for examining bovine patients with orthopedic issues that involve swelling of the distal limb at the University of Veterinary Medicine’s University Clinic for Ruminants.

After obtaining a complete history of each patient, a clinical and orthopedic examination including locomotion scoring according to Sprecher et al. (1997) was performed [20]. The patients were restrained on a tilt table in lateral recumbency to examine the affected limb including the claws. After performing functional claw trimming on all claws, the hair of the affected limb was clipped from the coronary band up to the proximal third of the metacarpal or metatarsal bones. The leg was cleaned and all clinical findings regarding the digit and leg were recorded. If wounds or claw horn disruption lesions were present, a probe was inserted to assess their depth and extent.

In all patients, the affected region was examined ultrasonographically using a 5–7.5 MHz linear probe. A protocol was followed that included the examination of all three digital joints, the DFTS of the neighboring digit, and all relevant bone surfaces. The entire digit was scanned in a proximodistal, dorsoplantar or -palmar, and lateromedial direction, both transversally and longitudinally [13,21]. The echogenicity of contents and the presence or absence of flow phenomena in the DFTS were used to determine the type of effusion. In the case of fibrinous tenosynovitis, as opposed to serous, serofibrinous or purulent effusions, no flow phenomena can be observed. In cases where the type of inflammatory effusion (serous, serofibrinous, fibrinous or purulent) could not be determined ultrasonographically by the clinician, affected synovial structures (DFTS or digital joints) were punctured and the synovial fluid was examined macroscopically.

Radiographic examination is not generally necessary to confirm the diagnosis of septic tenosynovitis of the DFTS. However, in cases where there was ultrasonographic evidence of involvement of the metacarpal, metatarsal, proximal sesamoid, or digital bones, or when there were large penetrating wounds, a radiographic examination was performed.

The final diagnosis was made after combining clinical, orthopedic, ultrasonographic, and other pertinent findings. After consideration of the general condition, age, and pregnancy status of the animal, a prognosis was given to the owner to facilitate their decision for treatment or euthanasia of the animal.

### 2.3. Methods of Treatment

#### 2.3.1. Preparing the Patients

All cattle were restrained in lateral recumbency on a tilt table with the affected digit above. Hind limbs were restrained with a strap proximal to the *tuber calcaneus* while front limbs were restrained proximal to the carpal joint. In cases where no digital amputation was performed, a therapeutic wooden or plastic block was applied to the sound partner claw. The distal limb was shaved, and the horn of the dewclaws was removed to a thickness of 1 mm. The limb including the claws and the interdigital space were diligently cleaned and disinfected.

All cattle received intravenous regional anesthesia [22]. A tourniquet was applied to the proximal metacarpal or metatarsal region or proximally to the tarsal or carpal joint. Local anesthetic (procaine hydrochloride, Procamidor^®^, 20 mg/mL, 400 mg, 20 mL, Richter Pharma AG, Wels, Austria) was injected into one of the digital veins, the lateral metatarsal vein or in the radial vein at the level of the carpal joint. One of the following surgical techniques was then used, depending on the diagnosis and the degree of involvement of adjacent anatomical structures.

#### 2.3.2. Incision of the Affected DFTS and Resection of the DFT (Group RDFT)

**Figure 1:**
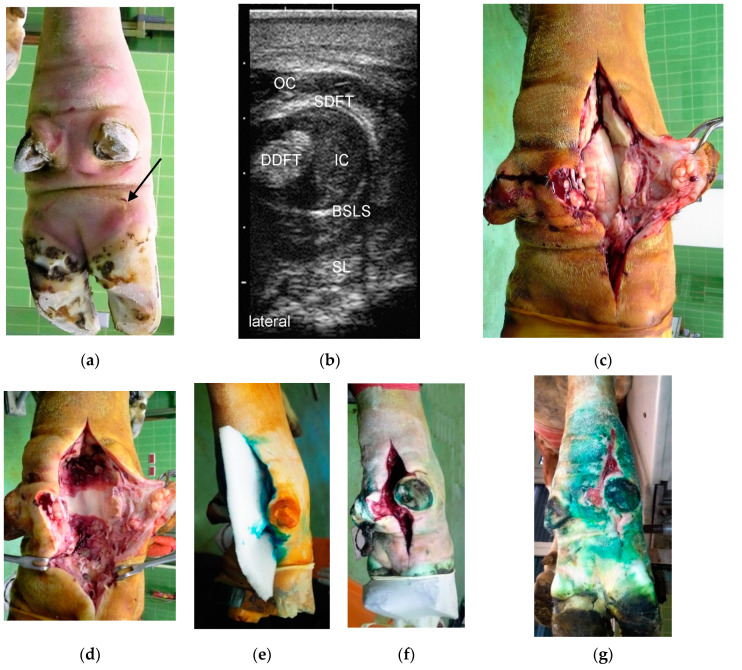
(**a**) Right hind limb of a five-year-old Fleckvieh cow with a four-day old penetrating wound (arrow), which only became apparent after clipping the hair; (**b**) Transversal sonogram (7.5 MHz linear probe) of the lateral DFTS approximately 2 cm proximal to the dewclaw with almost homogenously hypoechoic contents in both severely distended inner and outer compartments of the DFTS and no flow phenomena, indicating a fibrinous effusion. SDFT: superficial digital flexor tendon, DDFT: deep digital flexor tendon, BSLS: branch of the suspensory ligament to SDFT, SL: one of the suspensory ligament branches, OC: outer proximal compartment, IC: inner proximal compartment; (**c**) Intraoperative situation after incision of the lateral DFTS: the lumen is filled entirely with clotted fibrin; (**d**) Intraoperative situation after resection of both DFT, debridement and removal of all fibrin. The dorsal wall of the DFTS in severely hyperemic; (**e**) Use of sterile polyurethane soft foam as a drain; (**f**) Status three days after surgery during the first bandage change with a marked reduction in swelling and formation of granulation tissue; (**g**) Status at 12 days postsurgery with the wound almost entirely filled with healthy granulation tissue. Note that no part of the wound was sutured. After removal of the drain seven days post-surgery, the bandage facilitated satisfactory reduction of the width of the wound.

**Figure 2:**
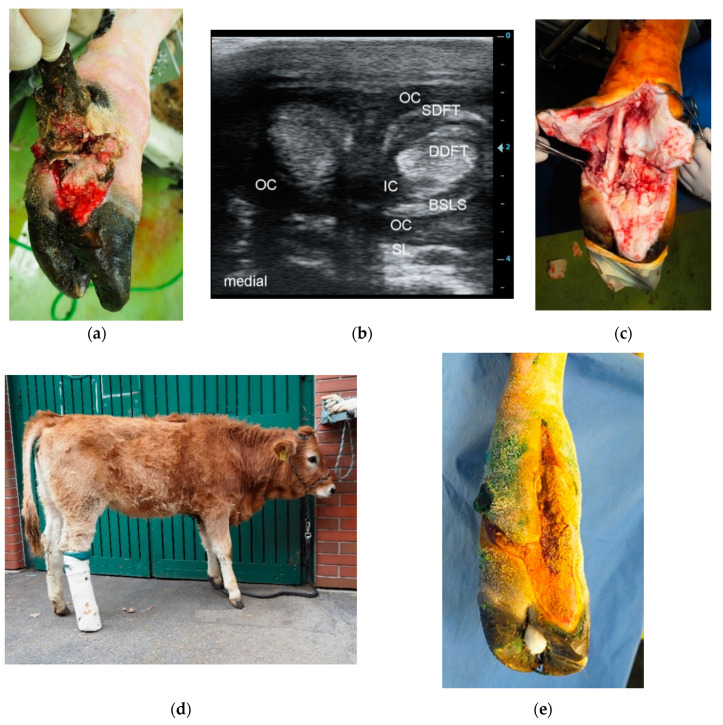
(**a**): Right hind limb of a seven-month-old Murbodner Blondvieh cross heifer with an eight-day old contused laceration across both DFTS with marked hypergranulation; (**b**) Transversal sonogram (7.5 MHz linear probe) of both DFTS approximately 1 cm proximal to the dewclaws showing marked distension, heterogeneous hypoechoic effusion with flow phenomena in the lateral DFTS indicating a purulent effusion and a mainly anechoic effusion with flow phenomena in medial DFTS indicating a serofibrinous effusion. SDFT: superficial digital flexor tendon, DDFT: deep digital flexor tendon, BSLS: branch of the suspensory ligament to the SDFT, SL: one of the suspensory ligament branches, OC: outer proximal compartment, IC: inner proximal compartment; (**c**) Intraoperative situation after opening of the lateral and medial DFTS, resection of both DFT of the lateral digit. The DDFT of the medial digit is visible where partial resection of tendon tissue was necessary. The entire medial DFTS was curetted and irrigated thoroughly; (**d**) The heifer eight days after surgery with a supportive bandage made of two PVC pipes; (**e**) Status at 14 days postsurgery with the wound almost entirely filled with healthy granulation tissue.

This method was chosen solely to treat cases of serofibrinous, fibrinous, and purulent tenosynovitis of the DFTS that were caused by penetrating wounds (Figure 1). The affected DFTS was incised from the proximal end (approximately 10 cm proximal to the dewclaws) to the distal end at the heel [16]. In most cases (*n* = 12 out of 16 in total), both the superficial and deep DFT were resected. The wound was carefully debrided and irrigated using 1000 to 2000 mL isotonic saline or diluted povidone-iodine solution (Vetisept^®^, 100 mg/mL, aniMedica GmbH, Senden-Bösensell, Germany). The DFTS was not closed; instead, a piece of sterile polyurethane soft foam (Ligasano^®^, Ligamed medical products GmbH, Cadolzburg, Germany) was inserted as both a drain and wound dressing (Figure 1d–f). Hyperextension of the affected claw was prevented as previously described [11], and a compression bandage was applied from the claw of the affected digit up to the proximal third of the metacarpal or metatarsal bone (Figure 2d).

#### 2.3.3. Incision of the Affected DFTS, Resection of the DFT, and Resection of the DIP Joint (Group RDIP)

In cases where septic tenosynovitis of the DFTS was diagnosed together with considerable infection of structures adjacent to the DIP joint, or when the DIP joint itself was observed to be affected by septic serous or serofibrinous arthritis, the affected structures were surgically removed, but the claw was spared (Figure 2) [11]. Wound management, including irrigation, drainage, prevention of hyperextension, and bandaging, was conducted as described above.

#### 2.3.4. Digital Amputation, Incision of the DFTS and Resection of the DFT (Group RAMP)

**Figure 3:**
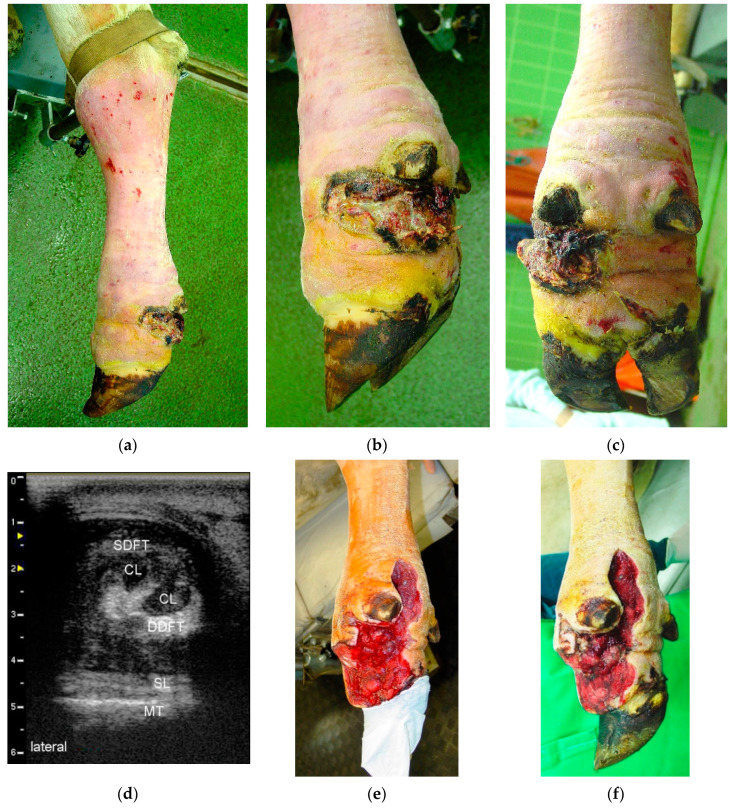
(**a**) Left hind limb of a three-year old Fleckvieh cow with the leg restrained for surgery; (**b**), (**c**) Lateral and plantar view of a 14-day-old wound above the lateral DFTS with marked hypergranulation and purulent exudate. Fibrinous-purulent tenosynovitis of the left lateral DFTS was diagnosed, as well as purulent tendinitis of both DFT and a purulent arthritis of the PIP joint; (**d**) Transversal sonogram (7.5 MHz linear probe) of the lateral DFTS approximately 2 cm proximal to the dewclaws showing heterogeneous hypoechoic effusion without flow phenomena and severe circumscribed loss of echogenicity of the DDFT as well as diffuse loss of echogenicity of the SDFT (CL: “core lesion“ caused by purulent disintegration of the tissue of the DDFT). There is marked effusion of the DFTS. SL: one of the suspensory ligament branches, MT: surface of the metatarsal bone. (**e**), (**f**) Status at 4 and 8 days postsurgery, respectively.

This method was applied where, in addition to septic tenosynovitis of the DFTS, the patient also had fibrinous or purulent arthritis of the DIP or PIP joint (Figure 3). The site of amputation was, depending on the extent of infection, either the middle of the second phalanx, in the PIP joint or in the distal third of the proximal phalanx [16]. Subsequently, the DFTS was incised and both DFT were resected [3,15,19]. Wound management, including irrigation, drainage, and bandaging, was conducted as described above.

In patients where septic serous or serofibrinous arthritis of the fetlock joint was diagnosed, the palmar or plantar recess of the joint was accessed via a 3 cm incision in the opened DFTS through two branches of the medial interosseus muscle [8]. The fetlock joint was flushed through and through with 3000 mL saline using a needle inserted dorsally into the synovial space.

#### 2.3.5. Peri- and Postoperative Medical Treatment

All patients were housed postoperatively in either a deep-bedded tie stall or a deep-bedded loose stall. Peri- and postoperative antibiotic treatment consisted of procaine penicillin and dihydrostreptomycin (Peni-Strepto^®^ 200/200 mg/mL, Virbac Laboratoires, Carros, France), ampicillin (Ampicillin “Vana”^®^ 200 mg/mL, 10 mg/kg i.m, Vana GmbH, Vienna, Austria), or oxytetracycline (Engemycin^®^ 100 mg/mL, 10 mg/kg i.m, Intervet GesmbH, Vienna, Austria) for three to ten days. Depending on their availability, these drugs were the standard antibiotic of choice for surgical interventions at different times at the clinic.

In some cases, these antibiotics were also applied to the opened DFTS. In other cases, tetracycline spray was applied to the wound. Ketoprofen (Rifen^®^ 100 mg/mL, 3 mg/kg i.m./i.v, Richter Pharma AG, Wels, Austria) was used as a nonsteroidal anti-inflammatory therapy [23].

The first bandage change occurred two to four days postsurgery under intravenous regional anesthesia to allow for pain-free removal of the wound dressing. The following bandage changes were conducted five to seven days apart. Starting with the second bandage change, Vaseline^®^ ointment was applied to the granulating wound to prevent attachment of the wound dressing to the granulation tissue.

The criteria for discharging patients from the University Clinic were advanced wound healing with complete coverage of the surgical wound with healthy granulation tissue (Figure 1g or Figure 2e or Figure 3f) and improvement of LS to a score of 2 out of 5 [20]. The owners were instructed to house the patients separately from the herd after their return to their farm of origin, to remove the bandage after five to seven days, and to irrigate the wound thoroughly with cold water twice a day for the next two weeks. After three weeks, the patients could be reintegrated into the herd and six to eight weeks after surgery, the owners were advised to remove the therapeutic block from the sound partner-claw.

### 2.4. Statistical Analysis

Statistical analysis was performed using Excel (Excel 2019, Microsoft Corporation, Redmond, WA, USA) and IBM SPSS Statistics for Windows, version 25.0 (IBM Corp., Armonk, NY, USA). Mean, standard deviation, and median were calculated using Excel. Using the Spearman rank correlation, possible associations between the age of patients, stage of lactation at the time of treatment and POST were explored. Because of patients that were still alive at the time of retrospective analysis, median cumulative POST using the Kaplan–Meier function was calculated.

## 3. Results

### 3.1. Patients

Patient records of a total of 83 cattle with septic tenosynovitis of the DFTS were reviewed. Seventy-five of the patients were female and eight were male. Of the female cattle, 47 were in lactation, 14 were dry, four were heifers that had not yet calved, and information regarding the lactation status of the remainder of the female cattle (*n* = 10) was not available. Most patients were Fleckvieh (*n* = 68) and Holstein Friesian (*n* = 11). Two Murbodner Blondvieh crosses, one Charolais, and one Pustertaler Sprinze were also included. At the time of admission to the University Clinic, the mean age of these 83 cattle was 4.3 years (±2.3; median: 4.4).

### 3.2. Clinical Findings, Diagnoses, and Localization

All cattle were examined clinically and orthopedically. In order to make a final diagnosis, an ultrasonographic examination was conducted in all patients. Radiographic examination was performed in 32 patients because there was ultrasonographic evidence of involvement of bones, or there were large penetrating wounds. Clinical and ultrasonographic examinations were complemented by a diagnostic puncture of the DFTS in 38 animals.

Overall, 104 cases of septic tenosynovitis of the DFTS were found in the 83 cattle. In 65 animals (78.3%), only one DFTS was affected (Figure 1 and Figure 3). In 16 patients (19.2%) two DFTS were affected (Figure 2) and three and four DFTS were affected in one animal each. Most frequently, lateral (*n* = 60; 57.7%) and medial (*n* = 21; 20.2%) DFTS of the hind limbs were affected. Only 23 cases (22.1%) of septic tenosynovitis of the DFTS were located in the fore limbs, almost uniformly distributed between medial (*n* = 11) and lateral (*n* = 12) digits. The mean LS of all patients was 3.5 out of 5 (±1.2; median: 4) at admission and 1.5 (±0.5; median: 1.5) at discharge (Table 1). Based on the character of the synovial effusion, we diagnosed serous (*n* = 10), serofibrinous (*n* = 18), fibrinous (*n* = 13), and purulent (*n* = 41) DFTS infections. In one case, the type of infection was not noted.

The cause of the septic tenosynovitis of the DFTS could be determined in most of the 83 patients. These were penetrating wounds in 32 cattle (Figure 1, Figure 2 and Figure 3) and ascending infections from claw lesions in 44 patients. In seven animals, we assumed the cause to be hematogenous infection based on the apparent lack of wounds or claw disorders.

Some patients had comorbidities such as bronchopneumonia (*n* = 2), septic polysynovitis (*n* = 1), valvular endocarditis (*n* = 1), septic arthritis of the tarsal joint (*n* = 2), septic precarpal bursitis (*n* = 2), right displacement of the abomasum (*n* = 1), and rupture of the serratus ventralis muscle (*n* = 1). Painful claw disorders diagnosed in these patients included interdigital phlegmon (*n* = 1), white line abscesses (*n* = 3), and sole ulcers (*n* = 4).

Depending on the different routes of infection in the 83 patients, other anatomical structures associated with the DFTS were affected. These were primarily the DFT (*n* = 53) and the DIP joint (*n* = 44), but also the fetlock joint (*n* = 16), the distal sesamoid bone (*n* = 16), the PIP joint (n = 12, Figure 3), the flexor tubercle of the distal phalanx (*n* = 9), the metacarpal or metatarsal bone (*n* = 3), the proximal sesamoid bone (*n* = 2), the tip of the distal phalanx (*n* = 1), and the digital extensor tendons (*n* = 1).

### 3.3. Methods and Duration of Hospitalization

Based on a poor prognosis, 28 of the 83 cattle were euthanized immediately after diagnosis. They suffered from severe infections affecting anatomic structures adjacent to the DFTS, affecting other limbs or organ systems. Some owners chose euthanasia based on the age of the patients or the cost of treatment. The remaining 55 cattle were treated surgically. In 16 patients (29.1% of treated animals), the DFTS was incised, irrigated, and one or more DFT were resected (group RDFT, Figure 1). Five animals (9.1%) were included in treatment group RDIP with additional resection of the DIP joint (Figure 2), and in 34 animals (61.8%), digital amputation was an additional part of the treatment (group RAMP, Figure 3). Because of septic serous or serofibrinous arthritis of the fetlock joint, in 16 patients an arthrotomy was performed and the joint was flushed through and through with saline.

Postoperative complications occurred in 17 of 55 patients (30.9%) (Table 2). These included the development of septic arthritis of the DIP joint (*n* = 6), wound infections (*n* = 5), infections of the DFTS in the neighboring digit (*n* = 3) or of the fetlock joint including the proximal sesamoid bone (*n* = 3). Of these 17 patients, 14 were treated by wound debridement, digital amputation, repeated irrigation of the DFTS of the neighboring digit through an incision from the existing wound of the primarily affected DFTS, and local and systemic antibiotic treatment. In the remaining three patients, euthanasia was carried out due to septic arthritis of the fetlock joint, persistence of severe lameness, and septic tenosynovitis of the neighboring DFTS of the same limb, resulting in poor prognosis and concerns regarding animal welfare. Two patients were euthanized later in the course of treatment at the University Clinic because they developed illnesses postoperatively unrelated to septic tenosynovitis.

The animals were treated systemically with antibiotics for an average of 6.2 days (±3.2) and for 3.3 days (±1.6) with nonsteroidal anti-inflammatory drugs. They were hospitalized for a median of 16 days (Table 1) and locomotion improved from moderate to severe lameness at admission (between score 3 and 4) to no or only mild lameness at discharge.

### 3.4. Postoperative Survival Time (POST)

Two patients were still alive at the time of retrospective analysis. The mean cumulative POST for all animals was 23.7 (±3.7) months. The reason for culling could be identified in 31 animals. Seven patients were culled because of problems related to lameness. Fertility issues were the cause for culling in ten animals, and nine patients were culled for various other reasons, for example, chronic mastitis or postpartum metabolic disease. Two animals were intended for fattening in the first place and went to slaughter and three animals of the RAMP group were culled precautiously because the owners were worried about future complications with only one claw on the treated limb.

The longest median survival time of 83.1 months was observed in group RDIP, which only included five animals (Table 2).

The age of the patients and the stage of lactation at the time of treatment had no significant effect on postsurgical survival time (*p* = 0.094 and *p* = 0.615, respectively). There was also no statistically significant difference with respect to POST between patients in groups RDFT and RAMP (*p* = 0.126). The number of cow in the RDIP was too low to make meaningful comparisons with the two other groups.

## 4. Discussion

In this study, we presented clinical findings, diagnostic approaches, and the outcome in cattle patients affected by septic tenosynovitis of the DFTS. In cattle that were subjected to treatment, we described three surgical methods, their associated postoperative complications, as well as their success, including improvement of locomotion and POST in a large number of cattle patients. To our knowledge, this study is the only retrospective analysis of postsurgical survival time in a large number of cattle after surgical treatment for septic tenosynovitis of the DFTS in the last 25 years.

The majority of cases of septic tenosynovitis of the DFTS in our study (60 out of 104 DFTS affected) were located in the lateral hind limb. This location is also most frequently affected by claw lesions, and ascending infections from those lesions were the main cause of infection of the DFTS in our study and others [3,5,19].

It has been described that cattle with septic tenosynovitis of the DFTS display lameness and swelling of the affected region to a varying degree, depending on the severity and duration of the process [3,10,19]. In our study, cattle had a median LS of 4 out of 5 at admission and all of them exhibited the typical distribution of (unilateral) swelling at the distal limb from the bulbs of the heel up to approximately 10 cm proximal of the dewclaw. In some cases, clinical and ultrasonographic findings were inconclusive, and the DFTS was punctured using a large bore needle to determine the nature of the effusion and help decide on further treatment and prognosis.

In several patients, in addition to the DFTS more structures of the digit were affected. Furthermore, in some animals, other organ systems were affected by various diseases causing a grave prognosis for recovery in those patients. This finding highlights the importance of performing a thorough clinical examination and a comprehensive ultrasonographic inspection of the complete distal limb region in animals presenting for the evaluation of lameness.

Different surgical methods were applied, depending on which adjacent structures were affected in addition to the septic tenosynovitis of the DFTS. Where penetrating wounds caused a primary infection of the DFTS, incising and irrigating the DFTS with or without resection of one or both of the DFT was the treatment of choice [3,10,15]. Where ascending infections from claw diseases were the cause of septic tenosynovitis of the DFTS, the additional resection of affected structures of the claw and, in most cases, additional digital amputation were necessary. The decision for choosing one of three surgical methods was based primarily on ultrasonography results. The decision between resecting the DIP joint and digital amputation was not solely based on medical necessity, but also on age and pregnancy status of the animal. The value of the animal and possible future production was weighed against the cost of treatment and the probability of recovery. It has been shown that the degree of lameness improves faster in cows with septic arthritis of the DIP after digital amputation compared with resection of the DIP joint. The cost of treatment is higher with joint resection, but the chance of developing disease of the partner claw is lower [24]. In our study, animals treated with RDIP remained at the clinic longest and the proportion of complications was highest compared to animals in the RDFT and RAMP groups.

In all patients included in this study, regardless of treatment group, the surgical wound was not closed, but left to heal by secondary intention, which is a method that has been previously described [3,15]. Closing a DFTS after removing fibrinous or purulent debris could lead to the retention of septic exudate.

An integral part of treating infected wounds is the use of peri- and postoperative systemic antibiotics. Highest priority critically important antimicrobials, such as third and fourth generation cephalosporins, should be avoided as drug of first choice, and, ideally, the antibiotic should be chosen after bacterial culture and sensitivity tests. However, this would cause a delay in treatment that interferes with animal welfare and the need for timely intervention. Therefore, we chose to treat the patients empirically or continued treatment that had been initiated by the referring veterinarians and used penicillin–streptomycin, ampicillin, and oxytetracycline for three to ten days as previously reported [4,6,25].

In cases that take an uneventful course, the wound can be completely covered by granulation tissue after two weeks [11,24,25]. Mean hospitalization periods of 26.5 (17 to 40) [3] and 24 (±15.1, 1–59) [25] days have been described after resection of the DFT and after digital amputation, respectively. In our study, cattle were hospitalized after these surgeries for a median of 12.5 days (1st and 3rd quartiles 11.0 and 16.5 days, respectively) and 16.0 days (1st and 3rd quartiles 14.0 and 21.5 days, respectively). At this point, despite the severity of the surgical intervention and the size of the wound, the cattle showed only mild lameness or were sound. Evaluating the duration of hospitalization as a measure of success of a treatment must be performed with caution because it is highly dependent on owner compliance and their ability to perform bandage changes at home.

It has been reported that cattle lived for 10 to 20 months [11,24,26] after resection of the DIP joint and resection of the DDFT at its insertion, and for 13 to 20 months after digital amputation, on average [17,18,25]. The range of POST as reported in these previous studies with surgical interventions of the same kind or similar severity is consistent with our findings. However, we also found that there is considerable variation in POST, which has also been shown in a study where resection of the DFT was performed. In this study POST was described as at least one to 69 months (mean 29 months) in 17 cattle. Nine of these animals were still in the herd at the time of the publication, and 15 cows had between one and six calves after treatment [3].

In our study, lameness was stated as primary reason for culling in only seven of 31 animals. However, other reasons, such as a reduction in fertility and metabolic disorders, might be associated with lameness and therefore represent a long-term consequence of septic tenosynovitis of the DFTS and the related treatment [27].

We could not show an influence of age and stage of lactation on POST in our study. However, it is important to note that the mean lifespan of dairy cattle in Austria is currently 6.3 years [28]. With a mean age at treatment of 4.3 (±2.3) years in our patients and an overall median POST of 15.5 months, the patients in our study were culled at an only slightly younger age compared to the average Austrian dairy cow.

## 5. Conclusions

In our study, most cases of septic tenosynovitis of the DFTS occurred in the lateral hind digit and were caused by ascending infections. Even though complications occurred postoperatively, 50 of 55 animals that underwent surgical treatment were discharged after a median hospitalization of 16 days. LS improved considerable during the time of hospitalization. POST was variable overall, but corresponded in part with previously reported data and demonstrated that treating animals is an economically reasonable opportunity to keep cattle in the herd. The animals appear to be able to cope well with one less claw and almost reach the life expectancy of an average Austrian dairy cow.

## Figures and Tables

**Table 1:** Median duration of hospitalization after surgery, locomotion score (LS) at admission and discharge and cumulative POST of cattle in the three treatment groups (1st and 3rd quartile).

Group	Hospitalization (d)	LS at Admission (out of 5)	LS at Discharge (out of 5)	Cumulative POST (Months)
All treated cattle	16 (12.75, 21.25)	4.0	1.5	15.5 (6.3, 34.0)
RDFT ^1^ (*n* = 16)	12.5 (11.0, 16.5)	3.0	1.0	17.3 (1.3, 31,5)
RDIP ^2^ (*n* = 5)	24.0 (18.0, 26.0)	4.0	1.5	83.1 (22.2, 109.0)
RAMP ^3^ (*n* = 34)	16.0 (14.0, 21.5)	4.0	1.5	11.9 (4.3, 23.9)

^1^ Resection of one or of both digital flexor tendons; ^2^ Resection of one or of both digital flexor tendons and resection of the DIP joint; ^3^ Resection of one or of both digital flexor tendons and digital amputation.

**Table 2:** Number of treated cattle and number of cattle with postoperative complications in the three treatment groups.

Group	Number of Treated Cattle	Number of Complications
RDFT ^1^	16	6
RDIP ^2^	5	3
RAMP ^3^	34	8

^1^ Resection of one or of both digital flexor tendons; ^2^ Resection of one or of both digital flexor tendons and resection of the DIP joint; ^3^ Resection of one or of both digital flexor tendons and digital amputation.
